# Periconceptional Non-medical Maternal Determinants Influence the Tryptophan Metabolism: The Rotterdam Periconceptional Cohort (Predict Study)

**DOI:** 10.1177/11786469241257816

**Published:** 2024-06-12

**Authors:** Sofie KM van Zundert, Lenie van Rossem, Mina Mirzaian, Pieter H Griffioen, Sten P Willemsen, Ron HN van Schaik, Régine PM Steegers-Theunissen

**Affiliations:** 1 Department of Obstetrics and Gynaecology, Erasmus MC, University Medical Centre, Rotterdam, The Netherlands; 2 Department of Clinical Chemistry, Erasmus MC, University Medical Centre, Rotterdam, The Netherlands; 3 Department of Biostatistics, Erasmus MC, University Medical Centre, Rotterdam, The Netherlands

**Keywords:** Tryptophan, kynurenine, serotonin, non-medical determinants, periconception period

## Abstract

**Background:**

The vital role of the maternal tryptophan (TRP) metabolism in maternal health and pregnancy is well established. However, non-medical maternal determinants influencing the TRP metabolism have been poorly investigated. We hypothesise that periconceptional maternal non-medical determinants alter the TRP metabolism, affecting both kynurenine (KP) and serotonin pathway (SP) metabolite concentrations. Therefore, we investigated the influence of non-medical maternal determinants on the TRP metabolism during the periconception period.

**Methods:**

About 1916 pregnancies were included from the Rotterdam Periconceptional Cohort between November 2010 and December 2020. Data on periconceptional non-medical maternal determinants were collected through questionnaires. Serum samples were collected at 8.5 (SD = 1.6) weeks of gestation and TRP, kynurenine (KYN), 5-hydroxytryptophan (5-HTP), 5-HT (5-hydroxytryptamine) and 5-hydroxyindole acetic acid (5-HIAA) were determined using validated liquid chromatography (tandem) mass spectrometry. Mixed models were used to determine associations between periconceptional non-medical maternal determinants and these metabolites.

**Results:**

In total 11 periconceptional non-medical maternal determinants were identified. Protein intake was positively associated with TRP (*β* = .12, 95% CI = 0.07-0.17), while age, energy intake and body mass index (BMI) (*β* = −.24, 95% CI = −0.37 to −0.10) were negatively associated with TRP. Age, BMI and total homocysteine were associated with higher KYN, whereas non-western geographical origin was associated with lower KYN (*β* = −.09, 95% CI = −0.16 to −0.03). Protein intake and total homocysteine (*β* = .07, 95% CI = 0.03-0.11) had a positive association with 5-HTP, while a negative association was found for energy intake. A non-western geographical origin and drug use were associated with higher 5-HT, and BMI with lower 5-HT (*β* = −6.32, 95% CI = −10.26 to −2.38). Age was positively associated with 5-HIAA (*β* = .92, 95% CI = 0.29-1.56), and BMI negatively.

**Conclusions:**

Periconceptional non-medical maternal determinants, including age, geographical origin, drug use, energy and protein intake, BMI and total homocysteine, influence KP and SP metabolite concentrations.

## Introduction

**Figure 1.**
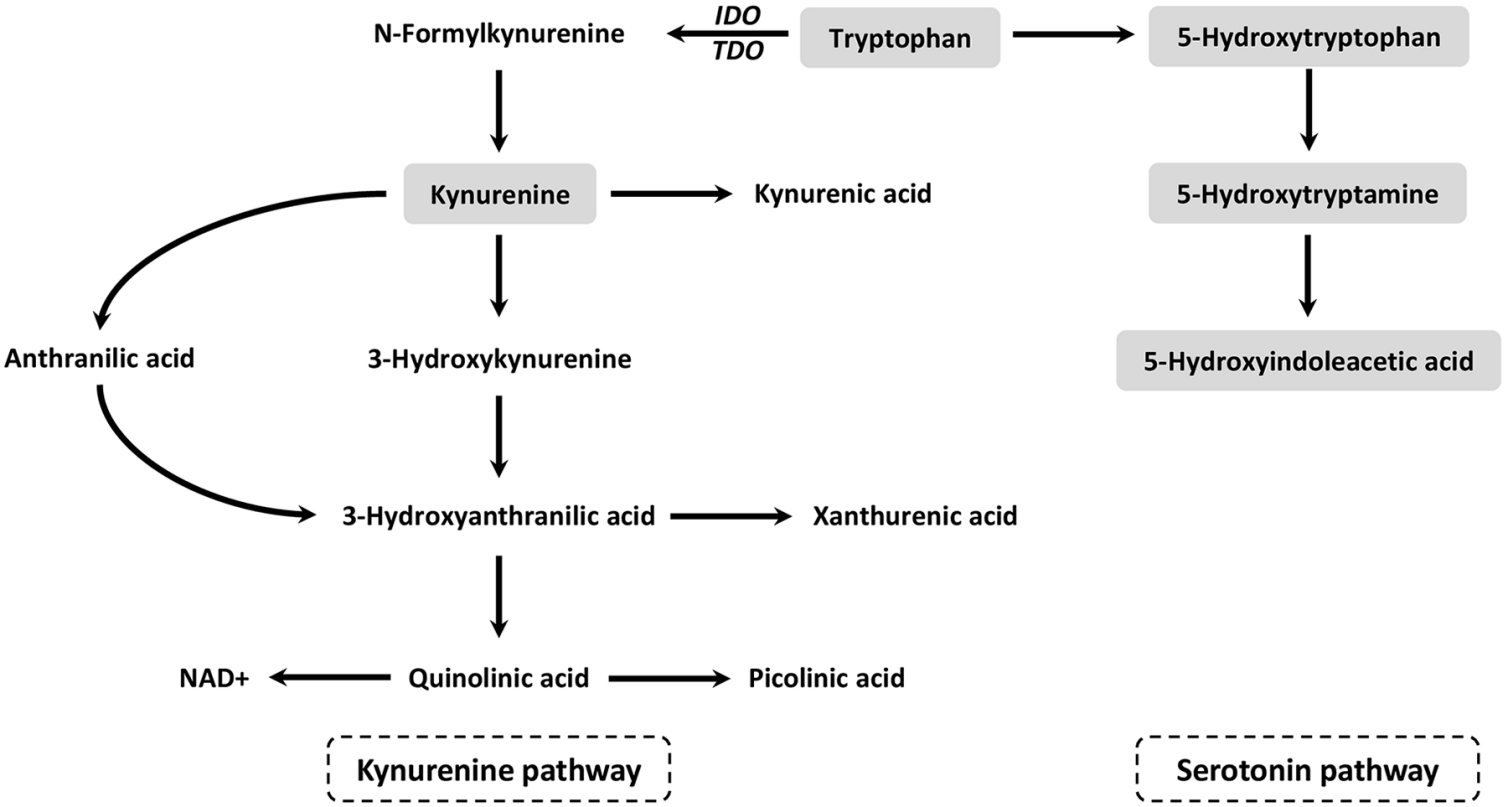
Kynurenine and serotonin pathway of the tryptophan metabolism. The analytes determined in this study are coloured grey. Tryptophan is converted to N-formylkynurenine by the hepatic tryptophan 2,3-dioxygenase (TDO) or the extra-hepatic indole amine 2,3-dioxygenase (IDO). This is the rate-limiting step in the kynurenine pathway. N-formylkynurenine is hydrolysed to kynurenine by arylformamidase. Kynurenine is further metabolised into the downstream kynurenine pathway metabolites kynurenic acid, anthranilic acid, 3-hydroxy-anthranilic acid, quinolinic acid, picolinic acid, and nicotinamide adenine dinucleotide (NAD+). Tryptophan is converted to 5-hydroxytryptophan, which is the rate limiting step of the serotonin pathway. Then, 5-hydroxytryptophan is converted to 5-hydroxytryptamine by aromatic amino acid decarboxylase. Finally, 5-hydroxytryptamine is converted to 5-hydroxyindoleacetic acid via 2 enzymatic steps involving the enzymes monoamine oxidase and aldehyde dehydrogenase.

Tryptophan is an essential amino acid required for protein synthesis, and is the precursor of many bioactive metabolites. Tryptophan is mainly metabolised along the kynurenine pathway (KP) in the liver (>95%) and the serotonin pathway (SP) in the gut and brain (Figure 1).^1,2^ The downstream metabolites of the KP play a role in vascular tone and immune modulation.^2-4^ SP metabolites are important for brain and gut function and are involved in the stress response.^5,6^ The effects of tryptophan metabolites can be both beneficial and harmful depending on the concentration and the cell type. A balanced tryptophan metabolism is important to maintain homoeostasis in cells and tissues. Indeed, alterations of the tryptophan metabolism have been associated with various non-communicable diseases, such as cancer, cardiovascular, and psychiatric diseases. For example, reduced tryptophan concentrations have been implicated in the pathogenesis of depression, increased kynurenine concentrations in coronary artery disease, and reduced serotonin concentrations in schizophrenia.^7-9^

Several non-communicable diseases originate during the periconception period, a critical period covering the 14 weeks before and the 10 weeks after conception. It involves essential metabolic processes that programme the developing embryo through epigenetic processes, with potential long-term health implications.^10^ Maternal exposures, such as poor lifestyle behaviours, including malnutrition, during this period are known to disturb several metabolic pathways, including the one-carbon metabolism.^10^ The one-carbon metabolism, comprising the interlinked folate and methionine cycles, and the transsulfuration pathway, is essential in providing one-carbon moieties for cell multiplication, cell differentiation and epigenetic programming.^10-12^ Homocysteine is a marker for derangement in one-carbon metabolism, which is one of the causal pathways that associates maternal determinants of health with an increased risk of impaired embryonic, foetal and placental development, and increased susceptibility to non-communicable diseases later in life.^10,13-18^ Derangement of the one-carbon metabolism often coincides with disruption of the tryptophan metabolism, as the tryptophan metabolism provides once-carbon units for the folate cycle and shares cofactors with the one-carbon metabolism.^10,19^ Tryptophan metabolites are involved in redox reactions, and can have pro- and anti-oxidative properties. Disruption of the tryptophan metabolism can induce excessive oxidative stress and subsequent inflammation, which are involved in adverse pregnancy outcomes.^20,21^ A body of evidence suggests that changes of maternal tryptophan metabolism, resulting in either an increase or decrease of the KP or SP, contribute to adverse pregnancy outcomes. Specifically, lower maternal tryptophan concentrations have been associated with depression during pregnancy, gestational diabetes, foetal growth restriction, and preterm birth, while higher kynurenine concentrations have been associated with gestational diabetes, and preterm birth.^22^ Additionally, there is evidence indicating that maternal tryptophan can modulate foetal brain development through placental synthesis of serotonin.^23^ However, our study specifically focussed on maternal tryptophan metabolite concentrations, and thus did not encompass an analysis of placental tryptophan metabolite concentrations.

Since the periconception period is a critical window of exposure, which shapes women’s and offspring’s health, increasing our understanding of maternal determinants that can influence the maternal tryptophan metabolism during the periconception period is of interest with regards to future prevention and early interventions of pregnancy complications and non-communicable diseases later in life. The influence of medical determinants on the tryptophan metabolism is widely acknowledged, especially concerning inflammatory diseases and medication use.^24,25^ In contrast, there are only a few studies that have assessed non-medical maternal determinants in relation to the tryptophan metabolism, with a particular scarcity of studies conducted during the periconception period. In this context, non-medical determinants refer to non-modifiable determinants such as age and geographical origin, as well as modifiable determinants such as lifestyle behaviours, and educational level. We postulate that periconceptional non-medical maternal determinants modify the tryptophan metabolism, leading to alterations of KP and SP metabolite concentrations. Therefore, the aim of this explorative study was to identify non-medical maternal determinants that increase or decrease KP and SP metabolite concentrations during the periconception period.

## Methods

### Study design and setting

This study was embedded within the Rotterdam Periconceptional Cohort (Predict Study), an ongoing prospective tertiary hospital-based cohort study performed at the Department of Obstetrics and Gynaecology of the Erasmus MC, University Medical Centre, the Netherlands (Erasmus MC).^26,27^ The Predict Study was conducted in accordance with the Declaration of Helsinki and approved by the Central Committee on Research in The Hague and the local Medical Ethics Committee of the Erasmus MC (15 October 2004, MEC-2004-277). Prior to inclusion, all participants provided written informed consent.^26,27^ Women and their partners were eligible to participate in the Predict Study if they had a child wish, and were at least 18 years old, as well as proficient in speaking and reading Dutch.^26,27^

### Study population

The current study focussed exclusively on women included from November 2010 to December 2020, that is, 2051 women with an average age of 32.4 (range: 18.0-48.6) years. Since this study focussed on tryptophan metabolites in serum collected during the first trimester of pregnancy, women were excluded from this study if they were not willing to provide a blood sample during this period. This resulted in a total study population of 1916 pregnancies.

### Non-medical determinants

The periconceptional non-medical maternal determinants investigated in this study were classified into 3 categories: non-modifiable determinants, modifiable lifestyle determinants, and other modifiable determinants (Table 1). Data on these determinants were collected using a general questionnaire and a validated food frequency questionnaire (FFQ) covering the periconception period, which were filled out before the intake appointment at the hospital. During the intake at the hospital at 8.5 (SD = 1.6) gestational weeks, anthropometric measurements were performed and blood was drawn.^26-29^ Maternal age was calculated at the moment of conception. Geographical origin was defined as non-Western or Western.^30^ Smoking, alcohol use, and drug use covered any substance used during the periconception period. Details regarding specific drugs were inquired, encompassing marijuana, amphetamine, XTC, cocaine, methadone, heroin, and unspecified other drugs. Energy intake (kilojoules) and protein intake (grams) were calculated per day from the FFQ. Folic acid supplement use was considered adequate if initiated before conception. Educational level was classified into low, middle and high based on the International Standard Classification of Education (ISCED).^31^ Body mass index (BMI) was calculated by the dividing the individual’s weight (kilograms) by the square of their height (metres). Total homocysteine (µmol/L) was measured in serum at 8.5 (SD = 1.6) gestational weeks as marker for lifestyle behaviours, including nutrition.^10^

**Table 1.** Periconceptional non-medical maternal determinants investigated in this study.

	Periconceptional non-medical maternal determinants
Non-modifiable determinants	Age at conception (years)
	Geographical origin (non-Western/Western)
Modifiable lifestyle determinants	Any smoking (yes/no)
	Any alcohol use (yes/no)
	Any drug use (yes/no)
	Folic acid supplement use (inadequate/adequate)
	Energy intake (kJ/day)
	Protein intake (g/day)
Other modifiable determinants	Educational level (low/middle/high)
	Body mass index (kg/m^2^)
	Total homocysteine (µmol/L)

### Tryptophan metabolites

Tryptophan (TRP) kynurenine (KYN), 5-hydroxytryptophan (5-HTP), 5-hydroxytryptamine (5-HT) and 5-hydroxyindole acetic acid (5-HIAA) were determined in serum, collected during the intake appointment at the hospital at 8.5 (SD = 1.6) gestational weeks, using a validated LC-MS/MS method.^32^ These metabolites will be abbreviated in the Results and Discussion sections.

### Statistical methods

Baseline characteristics were presented as means (standard deviation (SD)) or median (interquartile range (IQR)) for continuous variables depending on their distribution and as number of individuals (percentages) for categorical variables. Based on visual inspection of the distribution, the tryptophan metabolites were considered approximately normally distributed, except for 5-hydroxyindole acetic acid. Therefore, 5-hydroxyindole acetic acid was (natural) log transformed in all analyses. For the tryptophan metabolites a scatter plot matrix was created.

Mixed models were used to determine associations between periconceptional non-medical maternal determinants and tryptophan metabolites to account for possible correlations between pregnancies of the same women. Bivariable analyses were conducted for each determinant and tryptophan metabolite. Furthermore, a multivariable analysis was performed by including all determinants in a single model. The analysis was adjusted for gestational age at the blood draw.

To assess the robustness of the findings a cubic spline function was used to detect non-linear associations between the determinants and the outcomes and all two-way interaction terms were added to the multivariable model. Both did not improve the fit of the model, as indicated by higher or comparable Akaike information criterion (AIC) and Bayesian information criterion (BIC).

All statistical analysis were performed using R version 4.2.1.^33^ The results were presented as effect estimates with 95% confidence intervals (95% CI). A *P* ⩽ .05 was considered statistically significant.

## Results

### Baseline characteristics

#### Periconceptional maternal determinants

Table 2 displays the descriptive analysis of all variables. The average energy intake was 8350 kJ/day, of which 14.9% (73.3 g/day) derived from protein intake. Energy intake was comparable to the recommended daily intake (RDI) for energy (median (IQR): 9316 (8825-10 093) kJ) during the first trimester of pregnancy, while protein intake exceeded the first-trimester RDI by 14.2 g/day (median (IQR): 59.1 (52.8-68.3)), remaining within safe levels.^34,35^ The mean BMI was 25.8 (range: 16.5-53.0) kg/m^2^, which is just above the upper limit of normal (BMI normal range: 18.50-24.99 kg/m^2^).^36^ The average serum total homocysteine concentration in the first trimester of pregnancy was 6.1 (range: 2.5-53.7) µmol/L, and falls within the normal range of below 9 µmol/L.^10^

**Table 2.** Periconceptional non-medical maternal determinants and tryptophan metabolites of the total study population.

	Periconceptional non-medical maternal determinants	Total study population (*n* = 1916)
Non-modifiable determinants	Age at conception (y)	
	Mean (SD)	32.4 (4.6)
	⩾35 y	549 (28.7)
	<35 y	1367 (71.3)
	*Missing*	*0*
	Geographical origin^30^	
	Non-western	246 (14.2%)
	Western	1484 (85.8%)
	*Missing*	*186*
Modifiable lifestyle determinants	Any smoking	
	Yes	249 (14.5%)
	No	1470 (85.5%)
	*Missing*	*197*
	Any alcohol use	
	Yes	501 (29.1%)
	No	1218 (70.9%)
	*Missing*	*197*
	Any drug use	
	Yes	23 (1.3%)
	No	1695 (98.7%)
	*Missing*	*198*
	Folic acid supplement use	
	Inadequate, initiated after conception	328 (19.0%)
	Adequate, initiated before conception	1395 (81.0%)
	*Missing*	*193*
	Energy intake (kJ/day)^a^	
	Mean (SD)	8350 (2370)
	*Unreliable*	*343*
	*Missing*	*292*
	Protein intake (g/d)^a^	
	Mean (SD)	73.3 (19.9)
	*Unreliable*	*343*
	*Missing*	*292*
Other modifiable determinants	Educational level^31^	
	Low	132 (7.7%)
	Medium	616 (35.7%)
	High	978 (56.7%)
	*Missing*	*190*
	Body mass index (kg/m^2^)	
	Mean (SD)	25.8 (5.1)
	*Missing*	*45*
	Total homocysteine (µmol/L)	
	Median (IQR)	6.1 (2.0)
	*Missing*	*90*
Tryptophan metabolites	Tryptophan (µmol/L)	
	Mean (SD)	55.1 (9.6)
	*Missing*	*0*
	Kynurenine (µmol/L)	
	Mean (SD)	1.5 (0.4)
	*Missing*	*0*
	5-Hydroxytryptophan (nmol/L)	
	Mean (SD)	4.4 (1.6)
	*Missing*	*0*
	5-Hydroxytryptamine (nmol/L)	
	Mean (SD)	663 (286)
	*Missing*	*0*
	5-Hydroxyindole acetic acid (nmol/L)	
	Mean (SD)	49.1 (41.6)
	*Missing*	*0*

Continuous data are presented as means with standard deviation (SD) or median with interquartile range (IQR) depending on their distribution, and categorical data as numbers with percentages. The missing data per determinant are presented as numbers in italic.

a Calculated from the food frequency questionnaire (FFQ).

#### Tryptophan metabolites

**Figure 2.**
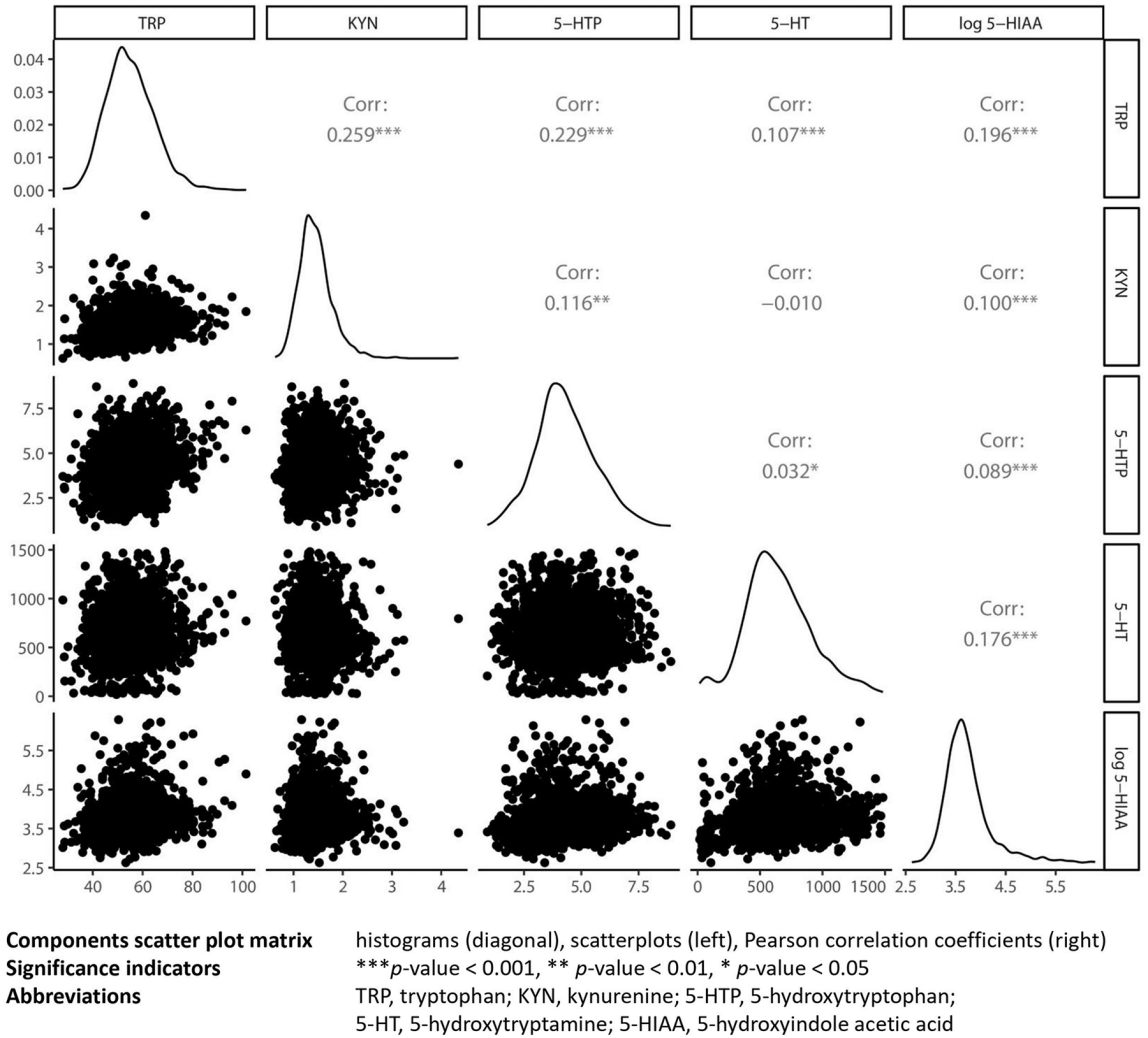
Scatter plot matrix of first-trimester tryptophan metabolite serum concentrations.

Descriptive analysis of the tryptophan metabolites and their correlations are presented in Table 2 and Figure 2, respectively. TRP was positively correlated with KYN, 5-HTP and 5-HIAA (*r* = .259, .229, .196, respectively), but less with 5-HT (*r* = .107). A weak correlation was found between KYN and 5-HTP and log 5-HIAA (*r* = .116 and .100), but not between KYN and 5-HT (*r* = −.010, *P* > .05). The SP metabolites were weakly, but positively correlated (5-HTP and 5-HT: *r* = .032, 5-HTP and log 5-HIAA: *r* = .089, 5-HT and log 5-HIAA: *r* = .176). All correlations, unless otherwise indicated, were statistically significant.

### Main results

**Figure 3.**
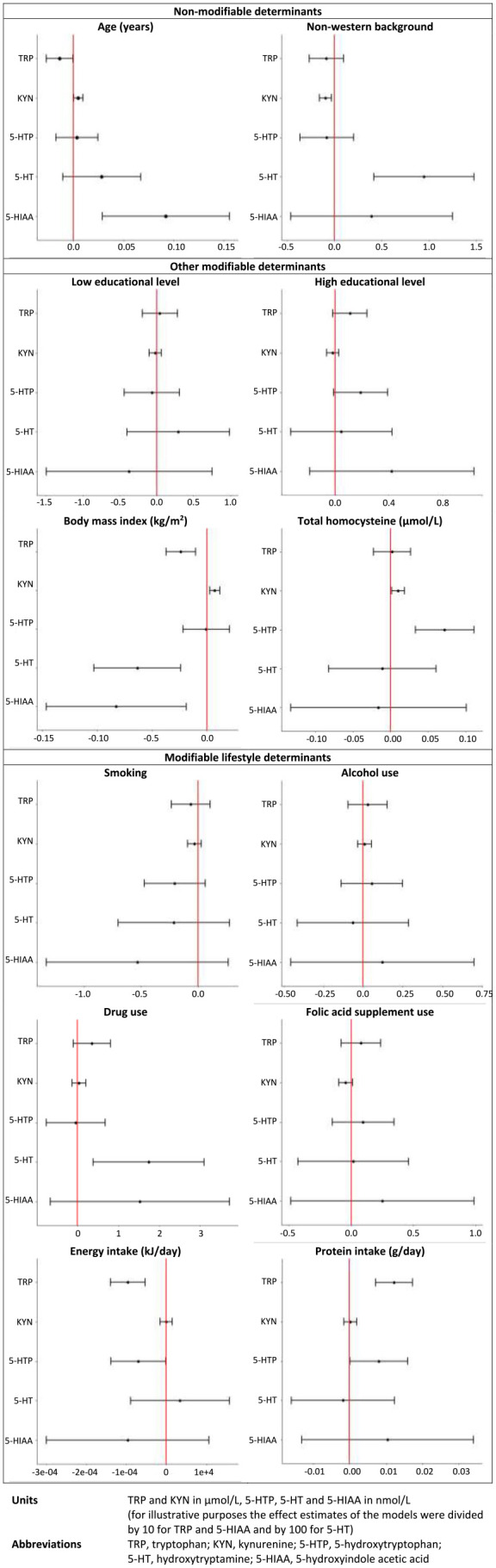
Forrest plots demonstrating the effect estimates including 95% confidence intervals of the multivariable mixed models for each determinant.

Supplemental Table 1 presents the results from the bivariable model and Table 3 from the multivariable model. The results of the multivariable analysis are described below and displayed in Figure 3. All associations mentioned in the Results section were statistically significant, unless otherwise specified.

**Table 3.** Adjusted associations between periconceptional non-medical maternal determinants and tryptophan metabolite concentrations in the first trimester of pregnancy.

Multivariable model		Tryptophan (µmol/L)		Kynurenine (µmol/L)		5-Hydroxytryptophan (nmol/L)		5-Hydroxytryptamine (nmol/L)		5-Hydroxyindoleacetic acid (nmol/L)	
		*β* (95% CI)	*P*-value	*β* (95% CI)	*P*-value	*β* (95% CI)	*P*-value	*β* (95% CI)	*P*-value	*β* (95% CI)	*P*-value
Non-modifiable determinants											
Age	Years	**−.14 (−.27 to −.01)**	.**041**	.**01 (.00 to .01)**	.**046**	.00 (−.02 to 0.02)	.745	2.82 (−1.07 to 6.71)	.153	.**92 (.29 to 1.56)**	.**005**
Geographical origin	Non- vs western	−.83 (−2.62 to 0.97)	.360	**−.09 (−.16 to −.03)**	.**005**	−.08 (−.36 to 0.20)	.578	**94.50 (41.78 to 147.22)**	.**001**	3.93 (−4.61 to 12.47)	.362
Modifiable lifestyle determinants											
Smoking	Yes vs no	−.63 (−2.33 to 1.07)	.462	−.03 (−.09 to 0.03)	.302	−.20 (−.47 to 0.06)	.134	−21.33 (−70.27 to 27.62)	.388	−5.33 (−13.34 to 2.69)	.189
Alcohol use	Yes vs no	.28 (−.95 to 1.51)	.649	.01 (−.04 to 0.05)	.702	.06 (−.14 to 0.25)	.570	−6.39 (−41.56 to 28.78)	.718	1.20 (−4.58 to 6.99)	.680
Drug use	Yes vs no	3.52 (−1.07 to 8.11)	.133	.04 (−.13 to 0.20)	.678	−.05 (−.77 to 0.67)	.897	**174.23 (38.34 to 310.12)**	.**012**	15.26 (−6.66 to 37.17)	.172
Folic acid supplement use	Adequate vs inadequate	.79 (−.79 to 2.37)	.320	−.04 (−.10 to 0.01)	.113	.10 (−.15 to 0.34)	.442	1.80 (−42.44 to 46.04)	.936	2.50 (−4.85 to 9.84)	.500
Energy intake	kJ/day	**−.00 (−.00 to −.00)**	<.**001**	−.00 (−.00 to 0.00)	.945	**−.00 (−.00 to −.00)**	.**045**	.00 (−.01 to 0.02)	.582	−.00 (−.00 to 0.00)	.347
Protein intake	g/day	.**12 (.07 to .17)**	<.**001**	.00 (−.00 to 0.00)	.867	.**01 (.00 to .02)**	.**047**	−.19 (−1.61 to 1.24)	.796	.10 (−.13 to 0.34)	.381
Other modifiable determinants											
Educational level	low vs medium	.42 (−1.95 to 2.80)	.723	−.02 (−.10 to 0.06)	.638	−.07 (−.44 to 0.31)	.728	29.14 (−40.06 to 98.34)	.404	−3.74 (−14.98 to 7.49)	.509
	high vs medium	1.10 (−.17 to 2.38)	.089	−.02 (−.06 to 0.03)	.430	.19 (-.01 to 0.39)	.065	4.70 (−32.93 to 42.32)	.804	4.20 (−1.89 to 10.29)	.173
Body mass index	kg/m^2^	**−.24 (−.37 to −.10)**	.**001**	.**01 (.00 to .01)**	.**003**	−.00 (−.02 to 0.02)	.963	**−6.32 (−10.26 to −2.38)**	.**002**	**−.83 (−1.46 to −.19)**	.**012**
Total homocysteine	µmol/L	.02 (−.23 to 0.27)	.880	.**01 (.00 to .02)**	.**022**	.**07 (.03 to .11)**	<.**001**	−1.08 (−8.26 to 6.09)	.765	−.16 (−1.33 to 1.01)	.785

The multivariable model included all non-medical determinants and was additionally adjusted for gestational age at the blood draw. Statistical significant results (*P* ≤ .05) are bold.

#### Non-modifiable determinants

Age was negatively associated with TRP and positively with KYN and 5-HIAA (TRP: *β* = −.14 95% CI = −0.27 to −0.01, *P* = .041; KYN: *β* = .01, 95% CI = 0.00-0.01, *P* = .046; 5-HIAA: *β* = .92, 95% CI = 0.29-1.56, *P* = .005). Age was also positively associated with 5-HTP and 5-HT, but not statistically significantly.

A non-western geographical origin was associated with lower KYN and higher 5-HT concentrations (KYN: *β* = −.09, 95% CI = −0.16 to −0.03, *P* = .005; 5-HT: *β* = 94.50, 95% CI = 41.78-147.22, *P* = .001). No other associations were found between geographical origin and tryptophan metabolites.

#### Modifiable lifestyle determinants

Figure 3 shows a clear decreasing trend of all tryptophan metabolites in women who smoked. No consistent trend was found with alcohol use. Drug use was strongly associated with 5-HT (*β* = 174.23, 95% CI = 38.34-310.12, *P* = .012), but not with other tryptophan metabolites.

Albeit not statistically significant, a positive association was found between folic acid supplement use and TRP and SP metabolites and a negative association with KYN.

Figure 3 illustrates the opposite effects of energy intake and protein intake. Energy intake was negatively associated with TRP and 5-HTP (TRP: *β* = −.00, 95% CI = −0.00 to −0.00, *P* = < .001; 5-HTP: *β* = −.00, 95% CI = −0.00 to −0.00, *P* = .045), while protein intake was positively associated with TRP and 5-HTP (TRP: *β* = .12, 95% CI = 0.07-0.17, *P* = < .001; 5-HTP: *β* = .01, 95% CI = 0.00-0.02, *P* = .047). Even though not statistically significantly, protein intake was positively associated with 5-HIAA in contrast to energy intake. No associations were found between protein nor energy intake and KYN or 5-HT.

#### Other modifiable determinants

Highly educated women had increased TRP and SP metabolite concentrations, while women with a low educational level had decreased 5-HTP and 5-HIAA concentrations compared to women with a medium educational level. However, none of these associations were statistically significant.

A negative association was found between BMI and TRP, 5-HT and 5-HIAA (TRP: *β* = −.24, 95% CI = −0.37 to −0.10, *P* = .001; 5-HT: *β* = −6.32, 95% CI = −10.26 to −2.38, *P* = .002; 5-HIAA: *β* = −.83, 95% CI = −1.46 to −0.19, *P* = .012). In contrast a clear positive association was found between BMI and KYN (*β* = .01, 95% CI = 0.00-0.01, *P* = .003). No such associations were found for 5-HTP.

Total homocysteine was positively associated with KYN and 5-HTP and not statistically significantly negatively associated with 5-HT and 5-HIAA (KYN: *β* = .01, 95% CI 0.00-0.02, *P* = .022; 5-HTP: *β* = .07, 95% CI = 0.03-0.11, *P* = < .001). No association between total homocysteine and TRP was found.

## Discussion

### Summary of results

**Figure 4.**
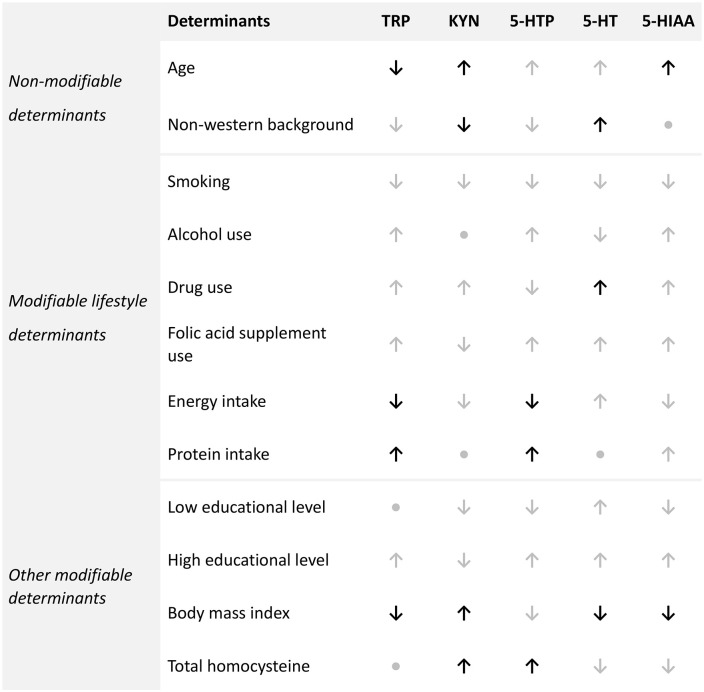
Summary of periconceptional non-medical maternal determinants of the tryptophan metabolism. ↑, positive association; ↓, negative association, •, no association. The associations that were significant in the multivariable model are coloured black. If the direction of the associations was the same in the bivariable as in the multivariable model but not statistically significant the arrows are coloured grey. Abbreviations: 5-HTP, 5-hydroxytryptophan; 5-HT, 5-hydroxytryptamine; 5-HIAA, 5-hydroxyindole acetic acid; KYN, kynurenine; TRP, tryptophan.

This study demonstrates that non-medical maternal determinants affected the tryptophan metabolism during the periconception period, as shown by increased and decreased KP and SP metabolite concentrations (Figure 4). The most pronounced and statistically significant associations are summarised in this paragraph. Age at conception was negatively associated with TRP and positively with KYN and 5-HIAA. A negative association was found between a non-western geographical origin and KYN, while a positive association was found with 5-HT. Drug use was strongly positively associated with 5-HT. Energy intake was negatively associated with TRP and 5-HTP, whereas protein intake was positively associated with TRP and 5-HTP. For BMI and total homocysteine, a positive association was found with KYN and with 5-HTP only for total homocysteine. BMI was negatively associated with TRP and SP metabolites.

Given the distinctions observed in tryptophan metabolism across species in earlier research, we prioritised human studies as our primary comparative reference.^37,38^ In instances where human data were lacking, we utilised relevant data from animal studies.

### Non-modifiable determinants

Age at conception – within the reproductive time-span – was positively associated with KYN and 5-HIAA and negatively with TRP, which is in line with previous studies investigating the effects of ageing on the tryptophan metabolism.^39-45^ Ageing is associated with an increasing pro-inflammatory status. This can cause upregulation of indole amine 2,3-dioxygenase (IDO) (Figure 1), leading to decreased TRP and increased KYN concentrations.^41,46^ A proposed mechanism for the age-related increase of 5-HIAA, is a compensatory response to the diminished efficiency of transport mechanisms with increasing age.^44^

Our findings regarding the association between a non-western geographical origin and tryptophan metabolites are in accordance with 2 earlier much smaller studies reporting decreased KYN and increased 5-HT concentrations in Afro-Americans, Hispanic-Americans and Asians compared to Caucasians.^47,48^ The mechanisms that explain the changes of tryptophan metabolites between different geographical origins are still largely unclear, but could be related to variations in genetics, diets and gut microbiota.^49^

### Modifiable lifestyle determinants

Any smoking during the periconception period was negatively associated with KP and SP metabolites in our study, which is consistent with prior research on (chronic) smoking.^39,50^ Conversely, one small study (*n* = 35) found a positive association between smoking and 5-HT measured 15 minutes after smoking a cigarette.^51^ This is in line with prior studies in rats, which reported increased 5-HT concentrations after acute administration of nicotine and decreased 5-HT concentrations after chronic administration of nicotine.^52^ An inhibitory effect of smoking on IDO has been reported and subsequent decreases KYN concentrations.^53,54^ Even though, it is suggested that IDO activity is reduced by smoking, no increased TRP concentrations were found in the present study or in previous studies.^53,54^

Surprisingly, the present study showed no associations between any periconceptional alcohol use and tryptophan metabolites, possibly attributed to the relatively low levels of alcohol use among these women during the periconception period. Previous research on the effect of alcohol on tryptophan metabolites has mainly focussed on chronic alcohol use and demonstrated increased KYN concentrations via activation of tryptophan 2,3-dioxygenase (TDO).^55^

Evidence suggests that drug use can alter both the KP as the SP of the tryptophan metabolism.^55,56^ We found a strong positive association between any periconceptional drug use and 5-HT, but considering the small number of women who used drugs and the different types of drugs used, this result should be interpreted with caution. The most reported drug in our study was cannabis, which can potentially influence the serotonin synthesis by inhibiting IDO. This can result in increased availability of TRP for the SP.^57^

Total homocysteine was positively associated with KYN, which substantiates the positive correlation between total homocysteine and the KYN/TRP ratio observed in earlier studies.^41^ Previous literature has demonstrated that folate, as substrate in the one-carbon metabolism, and the tryptophan metabolism are intertwined, as the tryptophan metabolism supplies one-carbon units to the folate metabolism.^10^ This is demonstrated by the FIGLU test, a diagnostic tool for folate deficiency used in the past, which is also positive when tryptophan deficiency is present. The conversion of FIGLU into glutamic acid is facilitated by the coenzyme tetrahydrofolate, a derivate of folate which is reliant on tryptophan as one of the one-carbon sources.^10,58^ Folate deficiency can lead to derangements in the one-carbon metabolism, of which total homocysteine is a sensitive biomarker.^10^ Hyperhomocysteinaemia and alterations of tryptophan metabolite concentrations are both involved in oxidative and inflammatory pathways.^10,20,21^ In addition, a deficiency in shared cofactors and substrates essential for both the tryptophan metabolism and one-carbon metabolism may potentially explain the observed positive associations between total homocysteine, KYN, and 5-HTP concentrations. Vitamin B6 serves as an illustrative example as it is crucial not only for converting total homocysteine into cystathionine, but also for the conversion of KYN into downstream metabolites and for the conversion of 5-HTP to 5-HT.^10,19,59,60^ However, future research is warranted to elucidate the exact mechanism underlying the observed associations, and explore the role of various cofactors in this complex interplay between the tryptophan metabolism and one-carbon metabolism.

The opposite effect of periconceptional energy intake and periconceptional protein intake on TRP and SP metabolites is in agreement with a prior study including only nine participants.^61^ When digested from protein rich food, the essential amino acid TRP is taken up in the circulation. It is suggested that in response to an energy-rich meal insulin increases, which supports protein synthesis by facilitating amino acids into the cells, resulting in decreased TRP concentrations.^61,62^

### Other modifiable determinants

To the best of our knowledge, no previous studies have assessed the association between educational level as exposure and tryptophan metabolite concentrations as outcomes. The negative association of BMI with TRP and SP metabolites and the positive association with KYN in our study are in line with previous studies.^32,63^ Both increasing BMI and a lower level of education have been associated with chronic inflammation.^64,65^ Increased pro-inflammatory cytokines can upregulate IDO. Consequently, tryptophan may be directed towards the KP, leaving less tryptophan available for the SP.^2,63^

### Strengths and limitations

A major strength is the prospective observational study design, which allowed for comprehensive data collection on multiple periconceptional non-medical determinants using (validated) questionnaires.^28^ Furthermore, the sample size in this study is very large in comparison to previous studies, and noteworthy, particularly considering the complex process of determining tryptophan metabolites. Another strength is the use of an accurate and robust LC-MS/MS method to measure tryptophan metabolites from both the KP and SP, which has been validated in women in the first trimester of pregnancy.^32^ In fact, this is the first study reporting the absolute concentrations of tryptophan metabolites from both the KP and SP of almost 2000 women in the first trimester of pregnancy.

In the current study, total tryptophan was measured instead of free tryptophan, which is known to be readily available for het KP and SP. However, free tryptophan is difficult to determine and easily influenced by several external determinants, such as timing of the blood sampling, which were not optimal in our study.^66^ Moreover, 5-HT was only measured in serum, which may have affected the results by 5-HT leakage from platelets. Data on platelet count and 5-HT concentrations in other blood fractions, such as platelet-rich plasma, could have given more information about the 5-HT metabolism and is recommended for future research.^67^ Despite the interconnectedness of the tryptophan metabolites within the same biochemical pathway, they were analysed individually due to their weak correlations and distinctive functions. Inherent to the explorative character of this study, no corrections for multiple testing have been applied. To further validate our findings and explore additional non-medical determinants, our study should be repeated in a population-based periconceptional cohort study.

### Implications and future perspectives

The results of this study suggest that the maternal tryptophan metabolism could be a shared pathway underlying the associations between periconceptional non-medical maternal determinants and adverse health outcomes, including pregnancy complications. For example, maternal age and BMI contribute to the development of various pregnancy complications, such as gestational diabetes and preterm birth, yet the underlying biological mechanisms are still not fully understood.^68-70^ Proposed biological pathways include excessive inflammation and oxidative stress.^46,71^ This study demonstrated that as maternal age and BMI increase, tryptophan concentrations decrease, while kynurenine concentrations increase. Decreased tryptophan concentrations and increased kynurenine concentrations have both been associated with excessive inflammation and oxidative stress, and have been found in women with gestational diabetes and in those who deliver preterm.^2,21,22,71-73^ Gaining more knowledge of determinants that affect the tryptophan metabolism during the periconception period, allows for a broader perspective on how this metabolism affects multiple aspects of maternal and foetal health. Consequently, this offers opportunities for preventive and therapeutic interventions that target multiple health outcomes simultaneously. Moreover, early markers may be identified that indicate the onset of adverse maternal health outcomes or pregnancy complications, even before clinically relevant and costly health impairments occur.

To gain more insight into the activity of the KP, the LC-MS/MS method should be expanded by other downstream KP metabolites, and kynurenic acid and quinolinic acid in particular considering their associations with various psychiatric disorders.^74,75^ However, it is important to consider the challenges in LC-MS/MS method development associated with these downstream metabolites, such as analyte stability, sensitivity and selectivity. Additionally, investigating additional biological pathways that modulate the tryptophan metabolism, such as the ability of cortisol to upregulate IDO in the KP, could shed light on their potential role in the development of pregnancy complications. Understanding the role of tryptophan in the maternal-placental-foetal metabolism, can enhance our knowledge of the aetiology of pregnancy complications, warranting further investigation in future studies.

## Conclusion

This study is the first to identify multiple periconceptional non-medical maternal determinants influencing tryptophan metabolism along the KP and SP. Specifically, maternal age, geographical origin, drug use, energy intake, protein intake, BMI, and total homocysteine level during the periconception period were found to be associated with changes in tryptophan metabolites. The maternal tryptophan metabolism may serve as a common pathway linking periconceptional non-medical maternal determinants to adverse pregnancy outcomes. Notably, modifiable non-medical maternal determinants, may be potential preconceptional targets for preventing alterations in tryptophan metabolism, with significant implications for maternal health and pregnancy outcome.

## Supplemental Material

sj-docx-1-try-10.1177_11786469241257816 – Supplemental material for Periconceptional Non-medical Maternal Determinants Influence the Tryptophan Metabolism: The Rotterdam Periconceptional Cohort (Predict Study) Supplemental material, sj-docx-1-try-10.1177_11786469241257816 for Periconceptional Non-medical Maternal Determinants Influence the Tryptophan Metabolism: The Rotterdam Periconceptional Cohort (Predict Study) by Sofie KM van Zundert, Lenie van Rossem, Mina Mirzaian, Pieter H Griffioen, Sten P Willemsen, Ron HN van Schaik and Régine PM Steegers-Theunissen in International Journal of Tryptophan Research
