# Supplementary material for: Periconceptional Non-medical Maternal Determinants Influence the Tryptophan Metabolism: The Rotterdam Periconceptional Cohort (Predict Study)
Source: Int J Tryptophan Res. 2024 Jun 12;17:11786469241257816. doi: 10.1177/11786469241257816 (PMC11171438; doi:10.1177/11786469241257816)
Supplement: sj-docx-1-try-10.1177_11786469241257816 – Supplemental material for Periconceptional Non-medical Maternal Determinants Influence the Tryptophan Metabolism: The Rotterdam Periconceptional Cohort (Predict Study) [file sj-docx-1-try-10.1177_11786469241257816.docx]

**Supplemental Table 1.** Unadjusted associations between periconceptional non-medical maternal determinants and tryptophan metabolite concentrations in the first trimester of pregnancy.

| **Bivariable model** | | **Tryptophan**  **(µmol/L)** | | **Kynurenine**  **(µmol/L)** | | **5-Hydroxytryptophan**  **(nmol/L)** | | **5-Hydroxytryptamine**  **(nmol/L)** | | **5-Hydroxyindoleacetic acid (nmol/L)** | |
| --- | --- | --- | --- | --- | --- | --- | --- | --- | --- | --- | --- |
|  |  | ***β* (95%CI)** | ***p*-value** | ***β* (95%CI)** | ***p*-**  **value** | ***β* (95%CI)** | ***p*-**  **value** | ***β* (95%CI)** | ***p*-value** | ***β* (95%CI)** | ***p*-**  **value** |
| **Non-modifiable determinants** | | | | | | | | | | | |
| Age | years | 0.00 (-0.09 – 0.10) | 0.949 | -0.00 (-0.00 – 0.00) | 0.914 | 0.01 (-0.01 – 0.03) | 0.208 | **2.93 (0.10 – 5.75)** | **0.042** | **0.76 (0.34 – 1.17)** | **<0.001** |
| Geographical origin | non- vs. western | **-1.96 (-3.26 – -0.67)** | **0.003** | **-0.09 (-0.14 – -0.04)** | **<0.001** | -0.17 (-0.40 – 0.05) | 0.123 | **77.69 (39.26 – 116.12)** | **<0.001** | -0.80 (-6.84 – 5.24) | 0.794 |
| **Modifiable lifestyle determinants** | | | | | | | | | | | |
| Smoking | yes vs. no | -1.11 (-2.40 – 0.18) | 0.092 | -0.01 (-0.05 – 0.04) | 0.799 | -0.21 (-0.43 – 0.01) | 0.064 | -23.47 (-60.95 – 14.01) | 0.218 | **-7.07 (-13.01 – -1.12)** | **0.020** |
| Alcohol use | yes vs. no | 0.66 (-0.34 – 1.66) | 0.191 | -0.01 (-0.05 – 0.03) | 0.583 | 0.04 (-0.13 – 0.21) | 0.654 | -4.66 (-33.08 – 23.75) | 0.746 | 3.04 (-1.52 – 7.60) | 0.190 |
| Drug use | yes vs. no | 0.92 (-3.04 – 4.88) | 0.648 | 0.01 (-0.13 – 0.16) | 0.865 | -0.32 (-1.00 – 0.35) | 0.344 | **174.09 (58.66 – 289.53)** | **0.003** | 4.80 (-13.50 – 23.09) | 0.605 |
| Folic acid supplement use | adequate vs. inadequate | **1.96 (0.81 – 3.11)** | **0.001** | **-0.05 (-0.09 – -0.01)** | **0.026** | 0.16 (-0.04 – 0.35) | 0.120 | 11.21 (-21.42 – 43.85) | 0.498 | **6.99 (1.74 – 12.25)** | **0.009** |
| Energy intake | kJ/day | **-0.00 (-0.00 – -0.00)** | **0.038** | -0.00 (-0.00 – 0.00) | 0.975 | -0.00 (-0.00 – 0.00) | 0.183 | 0.00 -0.01 – 0.01 | 0.936 | -0.00 (-0.00 – 0.00) | 0.354 |
| Protein intake | grams/day | 0.02 (-0.01 – 0.05) | 0.163 | -0.00 (-0.00 – 0.00) | 0.999 | 0.00 (-0.00 – 0.01) | 0.652 | 0.06 -0.70 – 0.81 | 0.883 | 0.02 (-0.10 – 0.15) | 0.702 |
| **Other modifiable determinants** | | | | | | | | | | | |
| Educational level | low vs. medium | -0.50 (-2.30 – 1.29) | 0.581 | -0.03 (-0.09 – 0.04) | 0.456 | -0.08 (-0.39 – 0.22) | 0.592 | 12.56 (-39.99 – 65.11) | 0.637 | -5.23 (-13.49 – 3.04) | 0.213 |
|  | high vs. medium | **1.73 (0.76 – 2.70)** | **0.001** | -0.03 (-0.07 – 0.00) | 0.062 | **0.17 (0.00 – 0.34)** | **0.047** | 17.53 (-11.46 – 46.52) | 0.234 | **7.18 (2.66 – 11.70)** | **0.002** |
| Body mass index | kg/m^2^ | **-0.28 (-0.37 – -0.19)** | **<0.001** | **0.01 (0.00 – 0.01)** | **<0.001** | -0.00 (-0.02 – 0.01) | 0.833 | **-6.19 (-8.77 – -3.60)** | **<0.001** | **-0.98 (-1.36 – -0.60)** | **<0.001** |
| Total homocysteine | µmol/L | -0.04 (-0.24 – 0.16) | 0.690 | **0.02 (0.01 – 0.03)** | **<0.001** | **0.07 (0.04 – 0.11)** | **<0.001** | -3.24 (-8.98 – 2.50) | 0.267 | -0.24 (-1.12 – 0.64) | 0.587 |
